# Maternal Priming of Offspring Immune System in *Drosophila*

**DOI:** 10.1534/g3.119.400852

**Published:** 2019-11-04

**Authors:** Julianna Bozler, Balint Z. Kacsoh, Giovanni Bosco

**Affiliations:** Department of Molecular and Systems Biology, Geisel School of Medicine at Dartmouth, Hanover, NH, 03755

**Keywords:** immune priming, immunity, intergenerational, leptopilina heterotoma, leptopilina victoriae, PGRP-LB, transgenerational

## Abstract

Immune priming occurs when a past infection experience leads to a more effective immune response upon a secondary exposure to the infection or pathogen. In some instances, parents are able to transmit immune priming to their offspring, creating a subsequent generation with a superior immune capability, through processes that are not yet fully understood. Using a parasitoid wasp, which infects larval stages of *Drosophila melanogaster*, we describe an example of an intergenerational inheritance of immune priming. This phenomenon is anticipatory in nature and does not rely on parental infection, but rather, when adult fruit flies are cohabitated with a parasitic wasp, they produce offspring that are more capable of mounting a successful immune response against a parasitic macro-infection. This increase in offspring survival correlates with a more rapid induction of lamellocytes, a specialized immune cell. RNA-sequencing of the female germline identifies several differentially expressed genes following wasp exposure, including the peptiodoglycan recognition protein-LB (PGRP-LB). We find that genetic manipulation of maternal PGRP-LB identifies this gene as a key element in this intergenerational phenotype.

Organisms routinely encounter dangerous and unpredictable challenges in the environment. In the face of such hardship, an individual can employ several strategies to maximize its fitness: an organism might prioritize its own physical condition over the survival of its potential offspring. Alternatively, an organism may invest in its progeny by better equipping the offspring to mitigate the effects of specific environmental threats. These strategies are described as “selfish parental effects” and “anticipatory parental effects”, respectively. Anticipatory parental effects may act through intergenerational phenotypic plasticity, imparting a phenotypic alteration to offspring in response to an environmental cue. Such phenotypic alterations can take the form of the transmission of environmental cue, such as light intensity (Galloway and Etterson 2007), and information about more stochastic conditions may also be transmitted to one’s offspring (Agrawal 2002). Given the challenges of predation, it is not surprising that there are examples of intergenerational plasticity in offspring defense phenotypes in multiple species (Agrawal 2002; Agrawal 1999; Agrawal *et al.* 1999; Tollrian 1990). In these cases, offspring inherit the environmentally induced phenotype, even when raised in a control environment that lacked the initiating environmental cue (Agrawal *et al.* 1999).

The immune system, or more specifically the adaptive immune response, has long been considered the pinnacle of environmental memory and defense. However, the innate immune system in insects is more adaptive than originally thought (Pham *et al.* 2007; Christophides *et al.* 2002; Kumar *et al.* 2003; Rodrigues *et al.* 2010. Inheritance of a *plastic* immune response has been observed in limited instances as well: *C. elegans* have been shown to transmit viral immunity across generations through small RNAs (Rechavi *et al.* 2011), and several bee species can confer enhanced resistance to bacterial challenges from queen to offspring (Little *et al.* 2003; Hernández López *et al.* 2014; Sadd and Schmid-Hempel 2006; Sadd *et al.* 2005; Sadd and Schmid-Hempel 2007). Alternatively, some studies have identified immune priming as being negative, where immune genes are downregulated following a priming event. Though these studies are *within* an individual and not demonstrated to be passed on to the next generation, the observation raises the possibility that intergenerational downregulation may also occur (Coussens *et al.* 2013; Kraman *et al.* 2010; Wetzig *et al.* 1982). Collectively, these examples all focus on past experiences with a pathogen (or parasite), and raise the question: “Can intergenerational immune priming occur in the absence of parental infection?” One might imagine the benefits of such a process in an instance where a pathogen or parasite specifically challenges the pre-adult stages of an animal.

Such is the case of certain endoparasitoid wasps, which are a persistent threat to wild Drosophila species (Fleury *et al.* 2004). These predatory wasps are specialized to infect the early larval stages of Drosophila by depositing their embryo and immuno-modulatory venom components, and, in the case of successful infection, consume the host prior to host eclosion. This event sequence leads to wasp eclosion (Schlenke *et al.* 2007). Various defenses have evolved to cope with this type of macro infection. For instance, *Drosophila melanogaster* undergo numerous behavioral changes in the presence of parasitic wasps, which have been shown to be protective against wasp infection (Kacsoh *et al.* 2013). Therefore, it is a possibility that other defense mechanisms, perhaps in offspring, could be altered to enhance survival from parasitism.

A key defense against infection for *Drosophila melanogaster* is a cell-mediated immune response. A variety of Drosophila immune cells have evolved to perform specialized functions that ultimately work in concert to isolate or otherwise destroy an intruder (Bozler *et al.* 2017; Kacsoh *et al.* 2014). For example, the members of melanogaster subgroup have a cellular immune response capable of melanotic encapsulation of macroparasites (Salazar-Jaramillo *et al.* 2014). One such macroparasite frequently encountered in nature is the egg of a parasitoid wasp that is injected into the body of a larva or pupa. Following infection with a wasp egg, circulating plasmatocytes, responsible for general phagocytosis, encounter and recognize the wasp egg as foreign. Next, a signaling cascade triggers the differentiation, proliferation, and release of larger specialized hemocytes known as lamellocytes (Stofanko *et al.* 2010). Once mobilized, lamellocytes, together with plasmatocytes, encapsulate the wasp egg, isolating it from the host body. Melanin and reactive oxygen species are then released within the cellular capsule by the crystal cells. This release launches a chemical assault on the parasite, culminating in a successful immune response (Nappi *et al.* 1995).

In healthy larvae, circulating hemocytes originate from embryonic macrophages (Holz *et al.* 2003). These peripheral hemocytes reside and proliferate in sessile hemocyte islets, a segmented compartment between the cuticle and muscle of the body wall in the larvae (Honti *et al.* 2014). The peripheral cell population is divided between circulating hemocytes and the non-circulating islets. A secondary source of hemocytes is derived from the lymph gland, which is segmented into different regions that are specialized for hemocyte maintenance and differentiation. Upon the initiation of metamorphosis, the lymph gland breaks down and releases the stored hemocytes (Lanot *et al.* 2001; Markus *et al.* 2009). Upon wasp infection, the lymph gland primary lobe can disintegrate early and release both plasmatocytes and lamellocytes into the circulating hemolymph (Sorrentino *et al.* 2002). This early release of lymph gland hemocytes has long been considered the primary source of lamellocytes. Although still somewhat controversial, evidence exists that suggests both cell populations can participate in lamellocyte production by triggering plasmatocytes of both origins to differentiate into lamellocytes (Anderl *et al.* 2016; Avet-Rochex *et al.* 2010; Honti *et al.* 2010; Stofanko *et al.* 2010). This has led some to propose a “demand-adapted” hematopoiesis model (Anderl *et al.* 2016).

Approximately 48 hr post wasp-infection, a significant increase in circulating lamellocytes can be seen (Márkus *et al.* 2005). One might speculate that a faster reaction to parasitism, with a more rapid production of specialized immune cells, might increase the success of the immune response. In this study, we describe an intergenerationally inherited immune phenotype, arising from parental exposure to wasps. Adult Drosophila are under no threat from these parasitoids, making changes in their behavior, such as egg laying reduction, anticipatory in an effort to protect offspring (Kacsoh *et al.* 2019). Although the parental flies are not infected, we find that their offspring have accelerated lamellocyte production and an increased survival rate following wasp infection, demonstrating that the behavioral change in oviposition rate is not merely egg holding, but rather a behavioral change that confers offspring-based immunity to the parasitoid threat. We further explore transcriptional changes in the oocyte and larvae from wasp-exposed females, and identify a key gene, PGRP-LB, for this immune plasticity.

## Methods

### Fly husbandry and immunity

Flies were maintained on cornmeal-molasses media as detailed in (Guo *et al.* 1996, 49-59) at room temperature (∼20-22°). A detailed list of fly stocks used can be found in supplementary information (Supplementary File 1—Table S6). During experimentation flies were kept at 25° with a 12-hour light-dark cycle. For the wasp-exposed treatment, 40 female and 10 male flies cohabitated a vial with 20 female *Leptopilina heterotoma* wasps for four days. The unexposed treatment was identical less the female wasps. Wasps were removed prior to egg collection.

Embryo collection lasted for 24-hours and was conducted on molasses-based agar plates supplemented with yeast paste. First instar larvae were transferred to petri dishes containing cornmeal-molasses food. After 48-hours, larvae were either used for blood cell experimentation or wasp infection.

Larvae used for blood cell counts were either poked with a sterile needle (penetrating the cuticle (induced)), or underwent mock conditions (uninduced control). After recovery, larvae were transferred to food dishes for an additional 24-hours. In 20 μl of PBS, hemolymph was harvested from five larvae (in the case of PGRP-LB^RNAi^ and Matα-Gal4, three larvae were used per replicate) and placed in a hemocytometer (Incyto C-Chip Neubauer Improved, DHC-N01-5). Slides were coded and hemocyte number was quantified in a blinded fashion. Samples were prepared and counted by different experimenters to ensure an unbiased, blinded quantification. Three replicates were performed for these experiments, and 18 grids were counted for each replicate. Two-tailed *t*-tests were used for significance tests, and standard error is reported for the error bars of all hemocyte counts.

In the case of wasp infection, 30 larvae, along with five *L. heterotoma or L. victoriae* female wasps, were used for a three-hour-long infection. To determine encapsulation success, larvae were dissected and categorized as infected and free of melanotic capsule, or infected with melanotic capsule. Instances of no infection or double infection were not included in the analysis. Data from 20 larvae were collected for each replicate, with three replicates per condition per experiment. Replicate data were pooled and a Fisher’s exact test performed for statistical reporting. All p-values are reported in Supplementary File 1—Table S1.

### Infection rate assay

Wasp strains were maintained and grown on the wild type strain of *D. melanogaster* genome strain (14021-0231.36, Drosophila Species Stock Center) (Kacsoh and Schlenke 2012). Prior to the infection choice assay, female wasps were allowed to infect *D. melanogaster* genome strain larvae (mixture of 2^nd^ and 3^rd^ instar) for 1.5 hr.

Infection plates were constructed as described above, with the noted exception that 20 Histone-RFP larvae were added to a plate with 20 larvae of the specified genotype/treatment condition. The RFP strain was selected because the larvae are not morphologically distinguishable from non-fluorescent lines under a standard dissecting microscope. Therefore, infection data could be collected in a blinded fashion under non-fluorescent conditions.

Three female *L. victoriae* wasps were added to infection plates for 30 min: Female wasps taken from a specific training plate were paired per replicate (one wasp cohort use for treatment and control plate), in this way the wasps would have had similar training experiences immediately prior to the infection choice assay. Following the infection period, wasps were removed and larvae were dissected approximately 24 hr later to determine infection status. All 40 larvae from each plate were dissected, after infection scoring the genotype was determined under a fluorescence microscope, thus, infection rate was scored blindly. Four replicates of each experiment were performed; however, one CS replicate was excluded due to very high infection rate approaching 100%.

Infection rate was normalized within each plate by dividing the proportion of infected CS (non-RFP) larvae to the proportion of infected RFP larvae. Each replicate was then normalized to the paired unexposed control group. A *t*-test was performed on the ratio of ratios in R (version 3.0.2 “Frisbee sailing”).

### RNA sequencing and qPCR

Wasp exposed fly groups consisted of 40 female and 10 male Canton-S flies along with 20 female Lh14 wasps, in a standard food vial for four days. The unexposed treatment was identical with the exception of the female wasps. Prior to sample collection, oviposition reduction of at least 60% was observed for each wasp exposed replicate. 40 ovaries were dissected for each replicate, stage 14 oocytes were isolated; the remaining ovary tissue was collected for separate processing. Four replicates were collected for each sample tissue and condition. RNA was isolated in Trizol and with a miRNA easy Qiagen kit with on column DNase treatment. Samples were ribo-depleted prior to random priming and sequencing; paired end sequencing at a depth of 40 million reads was performed on the Illumina platform.

Downstream analysis used the Ensembl genome (BDGP6) (Aken *et al.* 2016). Reads were mapped to the reference genome in CLC, and differential expression analysis was performed with EdgeR. Transcripts were considered differentially expressed if there was a significant false discovery rate (FDR >= 0.05), and a log2 fold change of 1. Alternatively, sequencing reads were indexed to transcripts using Kallisto with 100 bootstraps (Bray *et al.* 2016). Downstream processing and statistical analyses used Sleuth (Pimentel *et al.* 2017). Due to the data handling in the Kallisto/Sleuth pipeline, the beta values are approximate fold change values. Beta values are presented on the natural log scale, therefore a cut off of 0.7 was used to determine differentially expressed transcripts. The choice to focus on transcript allowed for a more nuanced detection of transcriptional variation, meaning that Kallisto allows us to perform transcript-level and gene-level analysis while simultaneously using bootstraps to ascertain and correct for technical variation between biological replicates (Bray *et al.* 2016; Pimentel *et al.* 2017). Such an analysis presents limitations as well, possibly losing detection of differential gene expression where a small expression change could be diluted across multiple transcripts, given that specific transcripts, not just genes are examined. In future studies, it may be interesting to analyze the RNA-sequencing data through a different pipeline, as Sleuth is known to be a much more conservative pipeline when compared to other gene-based approaches, such as cufflinks/cuffdiff.

Genes with at least one differentially expressed transcript in either pipeline were included in the DAVID analysis (Huang da *et al.* 2009; Huang *et al.* 2009). Presentation of DAVID data has transformed beta values to the log2 scale for ease of comparison; however, the converted beta values are approximations and, in this context, should be used to observe general trends between the pipelines, rather than for quantitative comparisons.

### qPCR

cDNA was generated using the QuantiTect Reverse Transcription kit (Qiagen). A complete set of primers used for qPCR can be found in supplement (Supplementary File 1—Table S7). Differential gene expression was determined with the delta-delta-Ct method. Ovary and oocyte samples were normalized to actin mRNA levels.

30 larvae were used per replicate (four replicates in total per experiment). Samples were collected in Trizol 24 hr after induction or mock treatment. Larval samples were normalized to RpL32.

### Data availability

All relevant data are contained within the paper while supporting information files are contained on figshare, with the exception of the raw sequencing files, which are on NCBI Sequencing Read Archive, with the corresponding BioProject ID PRJNA587308. Supplemental material available at figshare: https://doi.org/10.25387/g3.10156082.

## Results

### Enhanced cellular immunity

**Figure 1:**
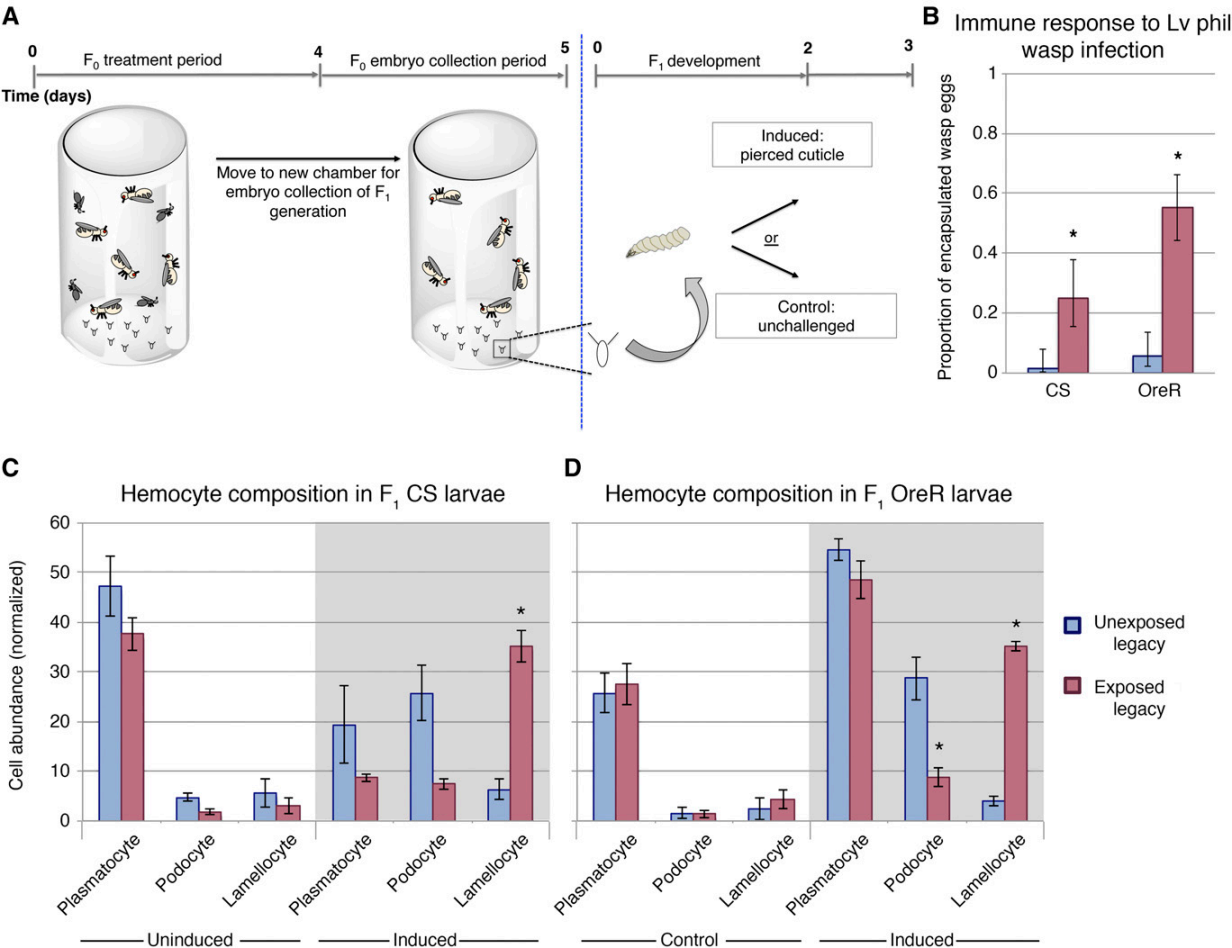
Exposed legacy offspring have enhanced cellular response following immune induction. Experimental design of intergenerational immunity effect (A). Wasp eggs are encapsulated at a higher rate in exposed legacy F1 larvae (B). Hemocytes were harvested and quantified from exposed legacy or unexposed legacy flies. Wild type lines Canton S and Oregon R were tested. Larvae from treatment groups had similar hemocyte composition under control conditions (C & D). Following immune induction, both Canton S and Oregon R exposed legacy larvae had altered composition and increased lamellocytes. Asterisk indicates p-value of <0.05.

To explore potential alterations in *Drosophila melanogaster* larval response to parasitism, we examined the hemocyte composition of two different wild-type genetic backgrounds, Canton-S and Oregon-R. We examined larvae from either wasp-exposed mothers (exposed legacy), or unexposed mothers (unexposed legacy). Under non-immune challenged conditions, both wild-type lines had similar levels of circulating lamellocytes. Immune induction can be achieved independent of wasp infection via a sterile needle mediated piercing of the larval cuticle (Márkus *et al.* 2005). Following sterile needle mediated piercing, we observe a significant increase in the number of circulating lamellocytes in exposed legacy larvae of both wild-type lines (Figure 1c, d, Supplementary File 1—Table S1), but not in subsequent generations in either line (Fig S1). It is important to note that we observe a general composition of hemolymph shift between induced and uninduced control, where we see a decrease in plasmatocytes and an increase in lamellocytes. Changes in immune cell abundance are expected following immune activation and agrees with previous studies describing the early immune response of *D. melanogaster* larvae (Anderl *et al.* 2016). Exposed legacy flies also exhibit a reduction in podocyte abundance when compared to unexposed legacy flies (Figure 1c, d). Although podocytes are not themselves directly involved in melanotic encapsulation, they are considered to be lamellocyte precursors (Stofanko *et al.* 2010). Therefore, one possible explanation for the reduced podocyte number is that these cells have already differentiated, or are differentiating, into lamellocytes.

The observation of enhanced cellular immunity in exposed legacy larvae translates into an increase in successful immune response against the wasp *Leptopilina victoriae* (Figure 1B). However, encapsulation of the wasp *Leptopilina heterotoma* was not successful by CS larvae, with 0% encapsulation in the unexposed legacy, and 2.6% in the exposed legacy larvae, recapitulating previous studies (Lynch *et al.* 2016). This is not surprising, as *L. heterotoma* venom is known to lyse circulating blood cells and trigger apoptosis in pro-hemocytes within the lymph gland(Chiu and Govind 2002; Rizki and Rizki 1994). The wasp venom of *L. heterotoma* renders cellular immunity ineffective, whereas *L. victoriae* venom acts to deglycosylate immune cell proteins, but not to destroy them (Mortimer *et al.* 2012), providing the basis for the observed differences.

Interestingly, in addition to increased immune priming in larvae, we observe a reduced rate of wasp infection in CS exposed legacy flies when infection was carried out in a heterogeneous population of larvae (Fig S2). Although we cannot rule out the possibility of behavioral defenses that reduce the likelihood of infection (such as tunneling deep within the food), or interruption of the infection process by rolling (Robertson *et al.* 2013), it is also possible that enhanced cellular immunity is protective as an infection deterrent by making the host appear less suitable to a parasitoid. Future studies may investigate the wasp ovipositor as a potential detector of the enhanced cellular composition in fly larvae.

### Transcriptional profile of oocytes

**Figure 2:**
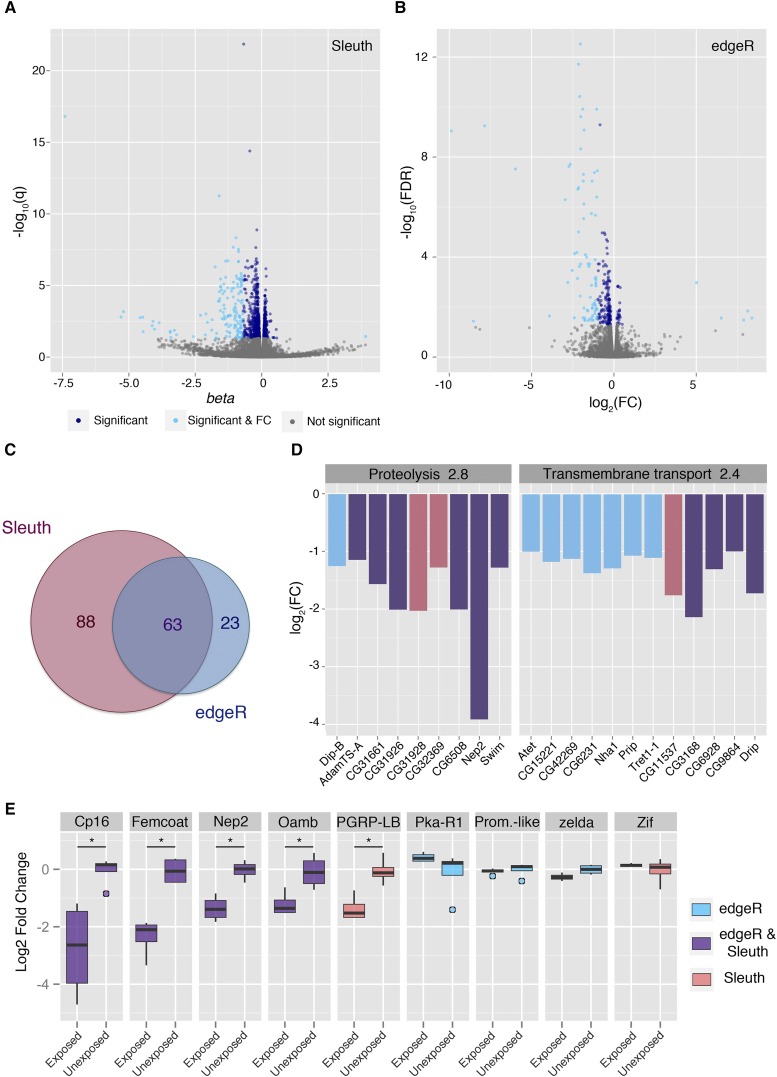
Transcriptional changes in stage 14 oocytes. RNA sequencing was performed on stage 14 oocytes and analyzed using two different methods: Volcano plots visualize differential gene expression for Kallisto/Slueth (A), and CLC/edgeR (B). Light blue points are differentially expressed transcripts, having both a significant test statistic as well as a log2 fold change of 2 or greater. Dark blue points are transcripts with a significant test statistic but falling below the fold change threshold. Venn-diagram displays the overlap between differentially expressed transcripts for the two analysis pipelines (C). DAVID functional annotation clustering is shown for the two most enriched clusters. Coloring indicates the pipeline that categorized the gene as differentially expressed (D). qPCR was performed to validate gene expression changes observed in sequencing (E). Asterisk indicates a p-value of <0.05.

To explore potential changes in the female germline and mature oocyte, we performed total RNA sequencing on stage 14 oocytes from wasp exposed or unexposed female flies. Analysis of the tissue with Kallisto/Sleuth identified 1067 transcripts as having a significant q-value. A subset of these transcripts was identified as differentially expressed by meeting a fold change threshold: 150 transcripts down regulated and one up regulated (Figure 2A, Supplementary File 1—Table S2). CLC/edgeR pipeline classified 264 as having a significant FDR, 81 of which were down regulated, and five up regulated (Figure 2B, Supplementary File 1—Table S3). In total, 62 transcripts were shared between the two pipelines (Figure 2C). This apparently low percentage of overlap between pipelines is not surprising and has been documented in significant detail(Soneson and Delorenzi 2013). For this reason, the two very different mapping tools were used.

Downstream functional cluster analysis with DAVID requires an input format of gene lists rather than transcripts. Therefore, a gene with any differentially expressed transcript was used in the source list for the cluster analysis. The stage 14 oocytes source list had 146 unique genes with at least one differentially expressed transcript. The functional analysis with these data returned 7 clusters with enrichment above 1. The two highest enriched clusters were annotated as proteolysis (2.8 enrichment), and transmembrane transport (2.4 enrichment) (Figure 2D).

Taking a candidate approach, we identified several potential transcripts of interest. Peptidoglycan recognition protein LB (PGRP-LB) is reported as a negative regulator of the immune deficiency pathway (IMD) and a regulator of the immune response to bacterial infection (Zaidman-Rémy *et al.* 2006). The IMD pathway in Drosophila plays an important role in the innate immune response as a mediator of bacterial infection. It is primarily implicated in innate immune recognition of gram-negative bacteria (Myllymaki *et al.* 2014). We find that PGRP-LB is down regulated in stage 14 oocytes, where Sleuth identifies a natural log fold change of -0.8 with significant q-value. Although edgeR does not identify a particular transcript of *PGRP-LB* to be differentially expressed in stage 14 oocytes, when considering gene abundance rather than transcript abundance, edgeR reports a log2 fold change of -1.12, with significant FDR. A subset of genes, including PGRP-LB, were selected for qPCR validation (Figure 2E); Cp16, Femcoat, Nep2, Oamb, and PGRP-LB were verified to have differential gene expression.

**Figure 3:**
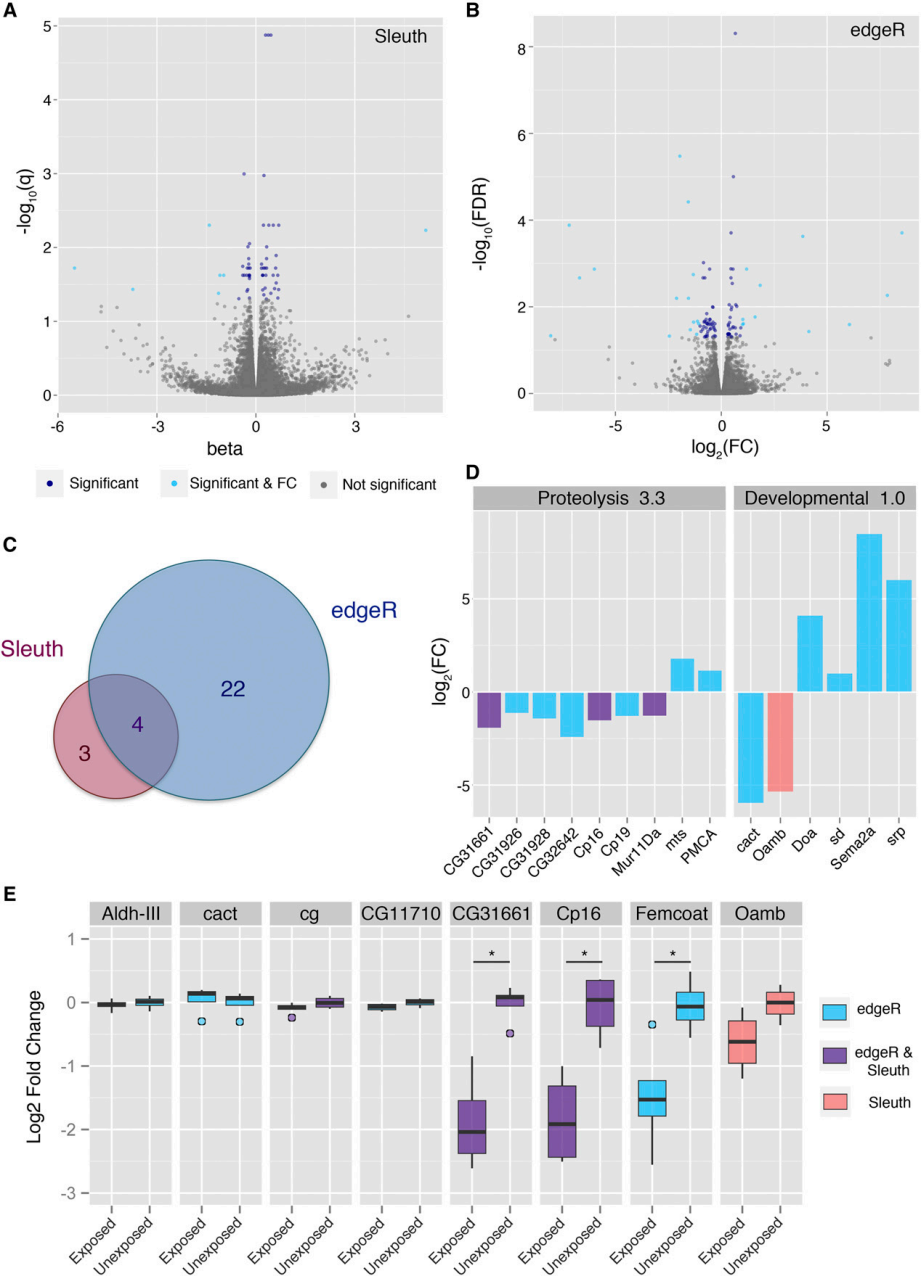
Transcriptional changes in ovary tissues. RNA sequencing was performed on ovary tissues and analyzed using two different methods: Volcano plots visualize differential gene expression for Kallisto/Slueth (A), and CLC/edgeR (B). Light blue points are differentially expressed transcripts, having both a significant test statistic as well as a log2 fold change of 2 or greater. Dark blue points are transcripts with a significant test statistic but falling below the fold change threshold. Venn-diagram displays the overlap between differentially expressed transcripts for the two analysis pipelines (C). DAVID functional annotation clustering is shown for the two most enriched clusters. Coloring indicates the pipeline that categorized the gene as differentially expressed (D). qPCR was performed to validate gene expression changes observed in sequencing (E). Asterisk indicates a p-value of <0.05.

Sequencing of ovary tissue lacking the stage 14 oocytes identifies minimal differential gene expression. Analysis of the ovary tissue with CLC/edgeR identifies 101 transcripts that had a significant false discovery rate (FDR). Of these, 24 transcripts meet the fold change cut off, with 15 down regulated and 11 up regulated transcripts. Alternatively, analysis with Kallisto/Sleuth identified 60 transcripts with a significant test statistic (q-value); seven of which meet the fold change cut off, with 6 down regulated and one up regulated transcript. The pipelines found agreement on four transcripts as differentially expressed (Figure 3A-C, Supplementary File 1—Table S4, Supplementary File 1—Table S5).

DAVID analysis returned two clusters with enrichment above 1, a peptidase and proteolysis group (3.3 enrichment), and development and alternative splicing group (2.0 enrichment) (Figure 3D). Although a cluster of immune related genes was not identified as enriched, the functional grouping identified are likely indicative of interesting and relevant biology, particularly in reference to the additional wasp-exposed phenotypes observed in female flies such as reduced egglaying (Lefèvre *et al.* 2012; Kacsoh *et al.* 2015). Genes in the proteolysis cluster are linked to diapause, or egg retention, in female flies(Baker and Russell 2009), and could be contributing to other behavioral phenotypes that have been previously described.

### Maternal PGRP-LB

**Figure 4:**
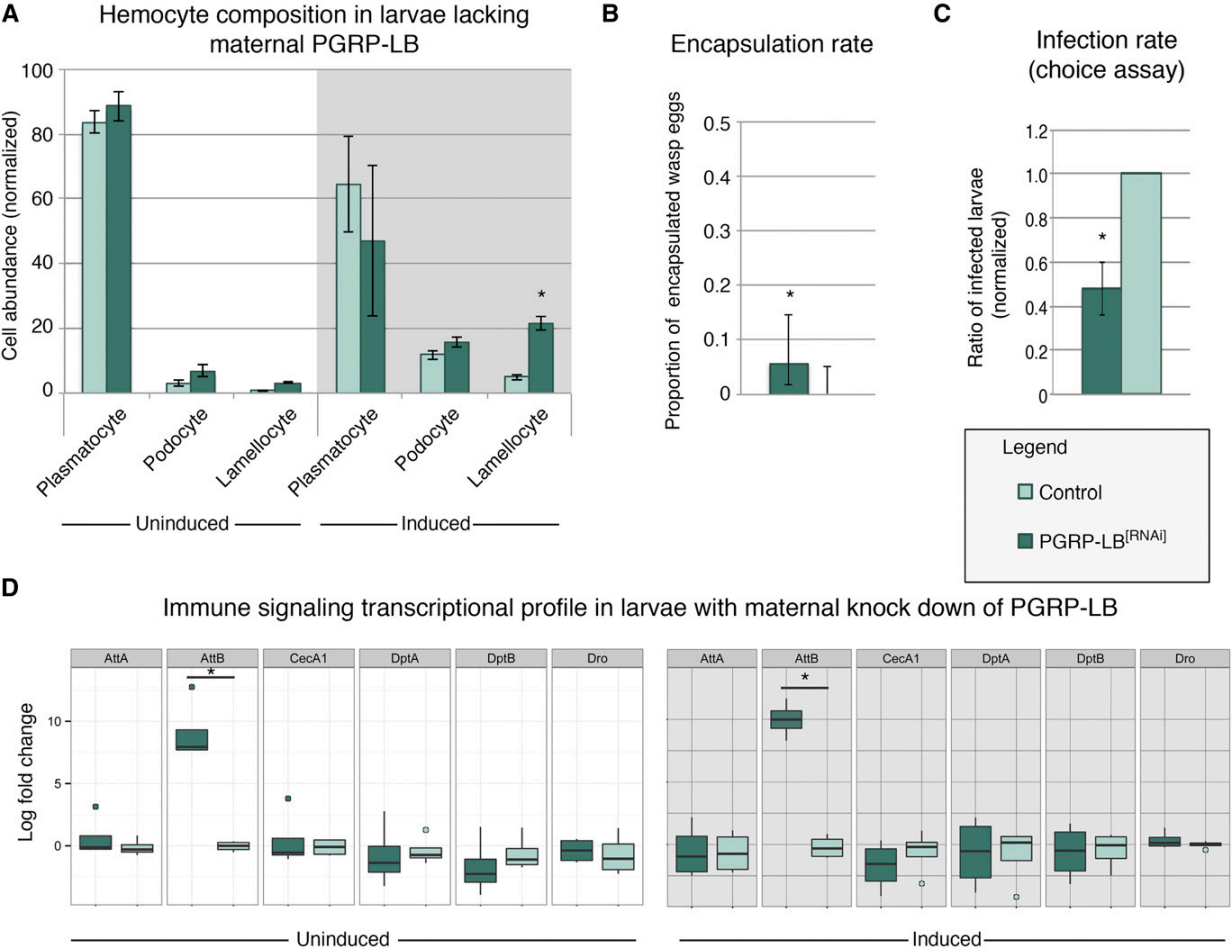
Larvae lacking maternal *PGRP-LB* have enhanced cellular response following immune induction. Hemocytes were harvested and quantified from larvae with maternal *PGRP-LB* germline knock down, or the Gal4 control line. Larvae of both genotypes had similar hemocyte composition under control conditions. Following immune induction, maternal *PGRP-LB* knock down larvae had altered composition and increased lamellocytes (A). Larvae from mothers with *PGRP-LB* knock down have elevated levels of *L. victoriae* wasp-egg encapsulation compared to the Gal4 control line (B), and reduced infection rates in the wasp infection-choice assay (C). qPCR of immune signaling genes (D). Asterisk indicates p-value of <0.05.

**Figure 5:**
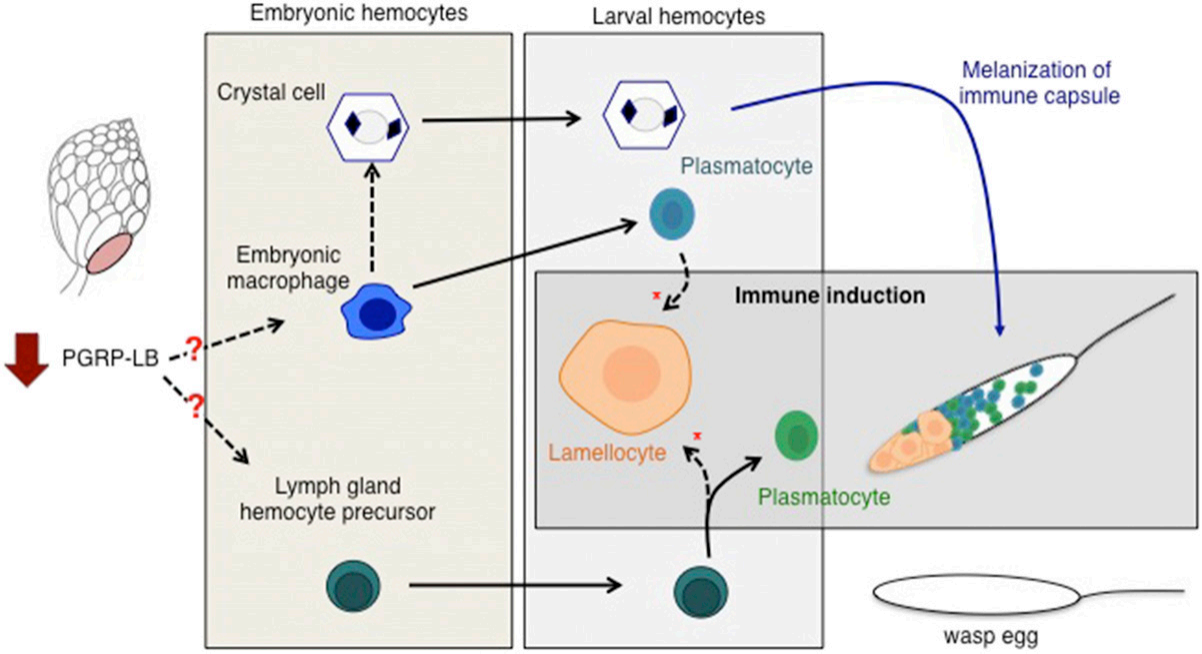
Model of hemocyte lineages during development. The two functional classes of embryonic hemocytes are the embryonic macrophage and crystal cells. Lymph gland hemocyte precursors, although present are not active until later in development. Hemocytes are able to differentiate and alter cell identity through known (solid line) and less-well characterized (dashed line) pathways. The late stage larva has three hemocyte types; plasmatocytes, crystal cells, and lamellocytes. Plasmatocytes originate from either the embryonic macrophage, or upon immune induction, from the lymph gland. Either source of plasmatocyte/prohemocyte can give rise to lamellocytes. All three cell types participate in wasp egg encapsulation. Exposure to wasps alters the dynamics of this process. We suggest that the maternal depression of *PGRP-LB* in the late stage oocyte impacts one or both of the embryonic tissues through an unknown mechanism (red question marks). This maternal signal alters the tissue(s) in a way that will enhance the differentiation of lamellocytes later in development (red asterisk).

Oocytes at stage 14 do not have zygotic transcription, and therefore, detected mRNA is maternally deposited during earlier oocyte development. To assess the potential role of *PGRP-LB* on the larval immune phenotype, we performed genetic knock-down of *PGRP-LB* using a maternal alpha-tubulin Gal4 (Matα-Gal4) driver specific to the female germline. Offspring from this genetic manipulation did not have significantly elevated circulating lamellocytes under uninduced control conditions. However, following immune induction, lamellocyte abundance significantly increased when compared to the *Matα-Gal4* driver line control (Figure 4A). Encapsulation rates against *L. victoriae* in larvae expressing *PGRP-LB* RNAi-transgene were elevated and statistically significant (Figure 4B). The observed increase in encapsulation rate is not as highly elevated as observed in wild-type larvae, suggesting a fundamental difference between maternal experience and genetic manipulation. This could be due to the efficacy of the RNAi-transgene, or suggestive of a yet-to-be identified negative regulator acting on *PGRP-LB*. Given previous characterization of the *Matα-Gal4*, we do not believe the finding is a result of GAL4 leakiness, as GFP expression is restricted to the ovary (Baskar *et al.* 2019). However, transgene expression elsewhere in the adult or embryo not examined may potentially yield a phenotypic effect. Furthermore, although we cannot exclude the possibility that the RNAi effect continues into early embryogenesis, we believe it unlikely that such effects would affect larval immune cell development. Nevertheless, such RNAi perdurance, if it were to occur, would likely be in addition to a maternal effect. Even with aforementioned alternatives, we find that infection rates were reduced in larvae with maternal *PGRP-LB* knockdown compared to the Matα-Gal4 driver line (Figure 4C). Collectively, these data suggest that maternally contributed mRNA is playing a *considerable* role in the inherited immunity phenotype of larvae, potentially by increasing the activity of the immune deficiency pathway or altering lamellocyte differentiation dynamics (Figure 5).

To evaluate the possibility that the increase in circulating lamellocytes is due to a global increase in immune signaling, we tested the expression of several key antimicrobial peptide genes, commonly used as markers for the NFκB pathway (Hetru and Hoffmann 2009; Ganesan *et al.* 2010). Of these genes, we find that AttB is elevated in both uninduced control and induced conditions (Figure 4D). However, a statistically significant increase in AttB mRNA levels was not observed in CS larvae (Fig S3), therefore the biological significance of this finding remains unclear.

## Discussion

In this study, we have presented a novel inherited immune phenotype in response to an environmental stimulus. This study is the third example of an inter- or transgenerational effect in Drosophila following wasp exposure (Bozler *et al.* 2019; Singh *et al.* 2015). Following a cohabitation period with a parasitoid wasp, parental flies generate progeny with increased cellular defenses. The production of lamellocytes is accelerated in these offspring, a phenotype that correlates with increased survival after wasp infection. Given the increased fitness advantage of a successful immune response, it is curious that lamellocyte production is not more rapid in wild type, unprimed larvae. This suggests that there are hidden costs associated with this phenotype, and that risk of parasitism must be high enough to induce enhanced immunity. A hyperactive immune system in *D. melanogaster* larvae has been described previously; in this case hyper-activation of the JAK/STAT pathway triggers self-encapsulation and melanotic masses are formed within the larvae in a devastating fashion (Rizki and Rizki 1980; Hanratty and Dearolf 1993). Perhaps the delay in lamellocyte formation is involved in an additional checkpoint to prevent such an assault. Additionally, a study that artificially selected for heightened immune defenses found that under resource-limited conditions, individuals with highly active immune systems experienced significantly reduced survival, demonstrating a clear trade-off in acquisition of a highly activated immune system (McGonigle *et al.* 2017). With considerable survival and fitness costs on either end of the immune response spectrum, it seems that phenotypic plasticity employs an “immune-Goldilocks” principle as an ideal natural adaptation, selectively priming the immune response only when chances of infection are high. Thus, offspring may achieve just the right response under the right conditions.

Further, we find that legacy exposed larvae experience reduced infection rates. Parasitoid wasps are known to sense and measure host suitability and infection status (Ruschioni *et al.* 2015; Bakker *et al.* 1967). These data raise the exciting possibility that parasitoid wasps may be capable of host selection based on immune competence. Although supposition, we believe this warrants additional investigation.

RNA sequencing of developed oocytes identified numerous differentially expressed transcripts. Our approach identified *PGRP-LB* as a candidate in inherited immune phenotype as it has been described as a negative regulator of the immune deficiency pathway (IMD) (Zaidman-Rémy *et al.* 2006). Further, *PGRP-SA* and *PGRP-SD*, two related immune-activating elements of the Toll pathway are upregulated following wasp infection (Schlenke *et al.* 2007). The IMD and Toll pathways regulate the expression of many of the immune related genes. Both pathways are required for a correct, and synchronized, immune response(Leclerc and Reichhart 2004; Kim and Kim 2005). Knock-down of maternal *PGRP-LB* mRNA is sufficient to recapitulate the inherited cellular phenotype, supporting our hypothesis and suggesting that this adaptive response is due to altered maternal loading of *PGRP-LB* into the oocytes. It is important to note that this RNAi transgene is not isoform specific, nor is the downregulated sequence detected by RNA sequencing a specific isoform. This observation suggests that global downregulation of *PGRP-LB* mRNA occurs, resulting in the inherited cellular phenotype.

It remains unclear how lamellocyte induction is accelerated. In other insects, it has been shown that both abundance and sensitivity of hemocytes can be modulated based on prior immune experiences (Rodrigues *et al.* 2010). Although we present a novel example of immune priming independent of an infection event, our data suggest that a similar process is occurring intergenerationally. It is possible that different pools of hemocytes are being utilized for this early response, as multiple origins of lamellocytes have been identified (Stofanko *et al.* 2010; Anderl *et al.* 2016) (Figure 5). Further, it is tempting to speculate that early embryonic tissues are being affected by maternal PGRP-LB, skewing them toward a more rapid lamellocyte differentiation. While questions remain regarding the up- and down-stream mechanisms, we have nonetheless identified a critical component in the inheritance of an environmentally triggered phenotype. Taken together, these various responses and pathways give a glimpse of how sensitive an organism can be to a single environmental condition: Only by analyzing the life history of the organism can this observation be uncovered. Drosophila immune priming now sets the stage for more molecular mechanistic studies that can provide insights as to how maternal germline cells sense environmental conditions and consequently respond to alter maternal contributions to developing oocytes. It will also be of great interest to determine whether Drosophila females sense general environmental threats and pass on general information, or instead whether specific sensing mechanisms produce unique outputs for coping with specific environmental threats.
